# Timely Communication Through Telehealth: Added Value for a Caregiver During COVID-19

**DOI:** 10.3389/fpubh.2021.755391

**Published:** 2021-11-29

**Authors:** Lauren Hajjar, Ben Kragen

**Affiliations:** ^1^Institute for Public Service, Suffolk University, Boston, MA, United States; ^2^The Heller School for Social Policy and Management, Brandeis University, Waltham, MA, United States

**Keywords:** telehealth (TH), care coordination, caregiver, chronic disease, Ehlers-Danlos Syndrome, relational coordination, health policy

## Abstract

**Objective:** This caregiver case study applies the lens of relational coordination theory (RC) to examine the value of telehealth as a medium of care coordination for a pediatric patient with hypermobile Ehlers-Danlos Syndrome (hEDS) during the COVID-19 pandemic.

**Background:** The COVID-19 pandemic has placed an unprecedented burden on the delivery of healthcare around the globe and has increased the reliance on telehealth services. Delivering telehealth requires a high level of communication and coordination within and across providers as well as between providers, patients and their families. However, it is less clear how telehealth impacts the coordination of care. In this paper, we provide insight into the quality of care coordination between providers and an informal caregiver following policy changes to the provider payment structure in Massachusetts.

**Methods:** This paper employs a single-case, autoethnographic study design where one of the authors uses their experiential insights, as mother of the patient, to inform a wider cultural and political understanding of the shift to remote caregiving for a pediatric patient with hEDS. Data was collected using reflective journaling, interactive interviews, and participant observation and analyzed using content analysis.

**Results:** Findings revealed four interrelating roles of the caregiver including, logistics support, boundary spanner, home health aide, and cultural translator. The adoption of telehealth was associated with improved timeliness and frequency of communication between the caregiver and providers. Findings about the impact of telehealth adoption on accuracy of communication were mixed. Mutual respect between the caregiver and providers remained unchanged during the study period.

**Conclusions:** This paper highlights areas where payer policy may be modified to incentivize timely communication and improve coordination of care through telehealth services. Additional insight from the perspective of an informal caregiver of a patient with a rare chronic disease provides an understudied vantage to the care coordination process. We contribute to relational coordination theory by observing the ways that caregivers function as boundary spanners, and how this process was facilitated by the adoption of telehealth. Insights from this research will inform the development of telehealth workflows to engage caregivers in a way that adds value and strengthens relational coordination in the management of chronic disease.

## Introduction

The COVID-19 pandemic has placed an unprecedented burden on the delivery of healthcare around the globe and has increased the reliance on telehealth services for remote care. Responding to such a complex and changing environment has required coordinated efforts between providers, payers, and consumers of healthcare to maintain patient safety and quality of care. One such effort took place in Massachusetts in March 2020 with the enactment of an emergency order which required insurers to reimburse telehealth delivered over video and phone at the same rate as in-person visits to ensure provider and patient safety (1). This payment policy shift allowed patients and their caregivers to access health and mental health services from the comforts of their home. However, delivering telehealth requires a high level of coordination within and across providers as well as between providers, patients and their families and more research is needed to understand how telehealth impacts the coordination of care among caregivers. In this paper, we provide insight into the quality-of-care coordination between providers and an informal caregiver of a pediatric patient following policy changes to the provider payment structure and service delivery method in Massachusetts. This case study looks at the use of telehealth and care coordination during the COVID-19 pandemic, a time when a large amount of care was shifted from in-person to remote delivery.

## Background and Literature Review

Care coordination has been identified by the Institute of Medicine (IOM) and the Agency for Health Research and Quality (AHRQ) as a key strategy in this effort to create value in health care (2). Care coordination can be understood as the organization of patient care activities between two or more roles involved in the delivery of healthcare services (3). It is an increasingly influential concept in health services research for its demonstrated ability in improving the effectiveness and efficiency of health care delivery (4). Care coordination is associated with a variety of performance outcomes including clinical outcomes, patient reported experience outcomes, and treatment adherence outcomes (4–7). Together these performance outcomes generate additional value for the health care system. Looking at ways to increase care coordination for children with complex chronic conditions, Golden and Nageswaran (8) noted a need for more information sharing and quality communication between caregivers and the rest of the clinical team.

Caregivers, typically family or friends, provide unpaid health care labor. They are increasingly being recognized for their contributions to patient care. Informal caregivers account for a large portion of the healthcare workforce in the United States. Approximately 65.7 million adults in the United States provided unpaid care to an adult or child in 2009 (9). On average these caregivers spent 20 hours each week providing care, totaling over a billion hours of informal care work each year in the United States (9).

Incorporating unpaid caregivers in care has been shown to contribute substantial value to the quality of care delivery. Informal caregivers help patients to make decisions about their treatment, making them an important stakeholder in the process of care delivery. Seminal medical anthropologists including Claude Levi-Strauss and Madeleine Leininger have long stressed the importance of incorporating patient's family and friends into the process of care delivery and their ability to illuminate aspects of the patient's personal and social life, such as dietary preferences or cultural practices, that must be taken into account when designing a treatment plan that works *in vivo* (10, 11). Nesting the treatment in the social lives of patients has been shown to improve critical measures like treatment adherence (12–14). The fact that caregivers are often unpaid and provide large amounts of labor means that they have the potential to generate quality without having to sacrifice efficiency, something that generates new value for the health care system. Telehealth is one mechanism that has been found to support the informal caregivers' role in health care delivery (15).

### Telehealth and Informal Caregivers

The COVID-19 pandemic has placed an unprecedented burden on the delivery of healthcare around the globe and has increased the reliance on telehealth services. Telehealth refers to the use of one or more electronic platforms to exchange health information, and is delivered by using synchronous video and audio-only technologies, as well as asynchronous messaging and remote patient monitoring. Generally, these platforms are accessed in one central location referred to as a patient portal. In response to the call for social distancing, Massachusetts policy leadership passed legislation that mandated reimbursement parity for the delivery of telehealth services for the duration of the pandemic (1, 16). The comprehensive legislation entitled, “An Act Promoting a Resilient Healthcare System that Puts Patients First”, broadly defines telehealth to include “the use of synchronous or asynchronous audio, video, electronic media or other telecommunication technology, including but not limited to, interactive audio-video technology, remote patient monitoring services, audio-only telephone and online adaptive interviews” (17). The new law addresses several important factors in making healthcare accessible including rate parity for primary care and chronic disease management telehealth services and increasing the scope of services for many specialists, including mental health providers.

Policy at the national level through the Office of Civil Rights has also increased the bounds of acceptable technology from Health Insurance Portability and Accountability Act (HIPPA) compliant technology to familiar applications like Apple FaceTime and Zoom (18). Another change to reimbursement policy was the development of virtual check-in codes by the Centers for Medicare and Medicaid Services, which allowed providers to be reimbursed for shorter appointments that occurred over the phone or through text-based secure messaging (19). Together these policy shifts enabled health care organizations to expand telehealth use by over 3,000 percent during the first month of the COVID-19 pandemic (20). Researchers are moving to study the effects of telehealth technologies on patient outcomes. This study observes the impact of this transition to remote care on care coordination with a caregiver of a patient with Hypermobile Ehlers-Danlos Syndrome (hEDS), a rare chronic disease.

Telehealth has been widely used by informal caregivers to aid in the delivery and coordination of care. Zulman et al. (21) found that 79% of respondents wanted informal caregivers to access some or all features of their patient portal. Of these respondents, 65%, 54% and 73% respectively indicated that they wanted to delegate communication with health care providers to a partner, family member, and unrelated caregiver respectively.

Tieu et al. (22) observed that informal caregivers generally report optimism about the ability of patient portals to support them as effective partners in care delivery. Telehealth can reduce critical barriers to care that are associated with in-person visits, such as transportation and child care (23). Researchers at the Veterans Health Administration found that a telehealth intervention designed to coordinate care has been shown to reduce hospital admissions by 19 percent, and bed days of care by 25% (24).

### Hypermobile Ehlers-Danlos Syndrome

Hypermobile Ehlers-Danlos Syndrome (hEDS) is an inherited chronic connective tissue disorder that primarily impacts the patient's skin and joints though can impact multiple systems in the body. It is common for patients to experience one or more of the following: joint hypermobility, early onset osteoarthritis, soft, velvety skin, variable skin hyper-extensibility, fragile skin with easy bruising, severe scarring and poor wound healing, musculoskeletal pain, arterial/intestinal/uterine fragility or rupture; scoliosis, poor muscle tone, mitral valve prolapse, and gum disease (25). The hEDS patient in this case experiences joint and skin related symptoms mentioned above in addition to co-occurring fatigue, gastrointestinal distress, dysautonomia, and anxiety.

Because hEDS can impact multiple systems in the body, patients are often referred to specialists for preventative screenings and/or to receive treatment depending on their symptoms. The genetics provider is considered a core member of the care team due to the inherited nature of hEDS. Other providers include: primary care, cardiology, orthopedics, rheumatology, physical therapy, gastroenterology, psychiatry, school nursing staff, counselor, teachers, etc. In the case discussed in this paper, each of these provider groups are associated with a different practice or hospital in various locations, with individual clinicians having varying degrees of knowledge of and experience working with hEDS patients. Connecting this disparate network is a feat, particularly during the COVID-19 pandemic, considering that many key stakeholders, like caregivers, do not operate within the healthcare system. Additional research is needed to understand how telehealth use impacts coordination of caregiving for patients with complex chronic diseases like hEDS. The aim of this study is to understand the impact of telehealth use on caregiving coordination during the COVID-19 pandemic.

This article is written as an autoethnography- a social science research method in which the corresponding author is a participant observer as an informal caregiver of a patient with hEDS. Autoethnography uses these experiential insights to inform a wider social and political understanding of a particular phenomenon (26). Two research questions guided our analysis: (1) How did the transition to telehealth services during the COVID-19 pandemic impact care coordination and inform the role of caregivers and quality of care and (2) What are the implications for health policy and practice?

## Theory

In 2007, AHRQ highlighted relational coordination as one of four frameworks that explained the relationship between care coordination and performance outcomes (3). Relational coordination (RC) is a framework derived from organizational theory and refers to a mutually reinforcing process of high quality relationships supported by high quality communication (27). Simply put, RC is communicating and relating for the purpose of task integration (28), and as such has been found to reduce the tradeoffs between quality and efficiency, pushing the quality and efficiency boundary outwards to generate new value.

**Figure 1:**
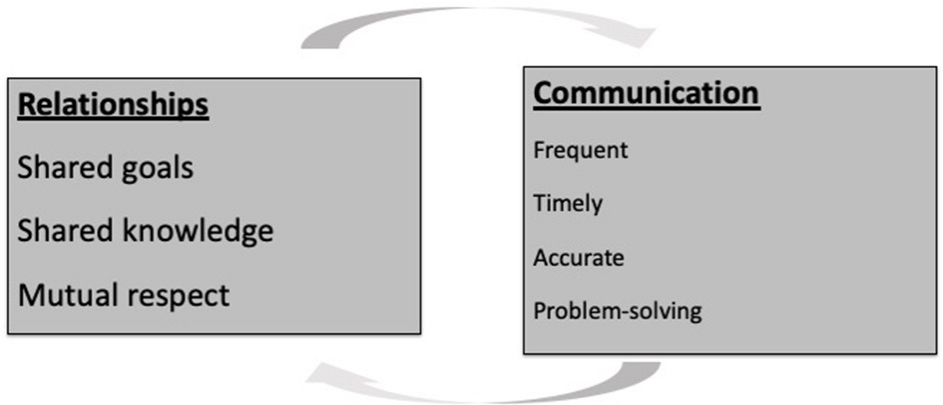
Seven dimensions of relational coordination. Relational Coordination is a mutually reinforcing process of high-quality relationships based on shared goals, shared knowledge and mutual respect and supported by sufficiently frequent, accurate, timely, and problem-solving communication. Source: Gittell (28).

As a construct, relational coordination consists of seven dimensions through which work is coordinated. Three relational dimensions include shared goals, shared knowledge and mutual respect. These relational dimensions are supported or reinforced by sufficiently frequent, timely, accurate and problem-solving communication and are expected to support a wide range of outcomes (29) (see Figure 1). For example, when individuals feel respected by others who are engaged in the same process, there is a tendency to experience higher quality communication. Likewise, when individuals share goals in a particular work process, they are more likely to engage in communication that is problem-solving, and less likely to blame others for poor performance. Finally, those who share knowledge about role responsibility are more able to engage in timely communication with one another as they understand what other roles need to contribute to the work process.

**Figure 2:**
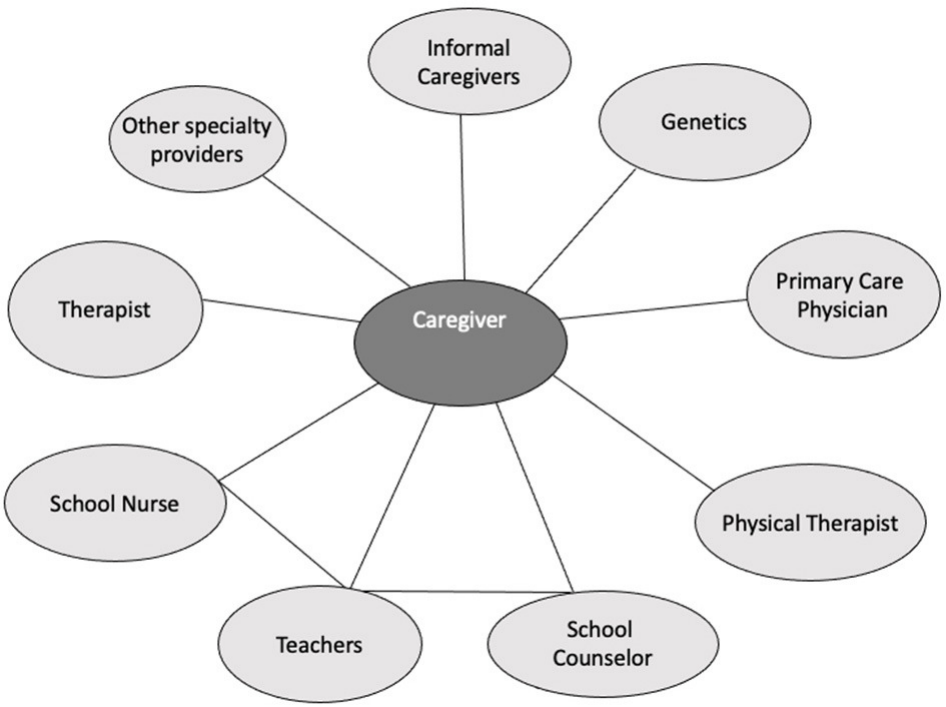
Network map of providers involved in caring for a pediatric patient with hypermobile Ehlers-Danios Syndrome (hEDS). The network map displays providers involved in the management of Hypermobile Ehlers-Danlos Syndrome (hEDS), including the informal caregiver. Source: Gittell (28).

Relational coordination been widely studied in health care with consistent results across organizations (29, 30). Strong RC across organizations serving the same constituents enables participants to achieve higher quality outcomes more efficiently (27, 28, 31, 32). A recent systematic review (29) identified several healthcare studies which positively associated relational coordination among interdisciplinary staff to quality outcomes including postoperative functional status, patient reported quality of care and quality of life, family satisfaction with care, patient trust and confidence with providers, and patient psychological well-being (27–29, 33–44). Despite the growing evidence of relational coordination on patient and provider outcomes, only 14% of all RC findings were based on relational coordination between providers and their clients, including caregivers (29). Thus, relative to its territory, RC remains under-explored between caregivers and providers. We extend the theory by examining RC between a caregiver and multiple providers treating a pediatric hEDS patient. In the network map above, coordination between providers is largely facilitated by the caregiver (see Figure 2).

RC theory (45) also predicts that certain organizational structures can support stronger relationships and communication within and across workgroups. For instance, boundary spanners can impact performance outcomes through their ability to facilitate relational coordination. Boundary spanners are broadly defined as or roles that are dedicated to coordinating between other roles (28, 46). Bragstad et al. (47) found that caregivers in-part function as boundary spanners, and generate performance outcomes and subsequent value for the health care system through their ability to mediate the relationship between the patient and their providers. Studies found relational coordination between patient's family members and care providers to be positively associated with high quality post-surgical outcomes, greater patient well-being and patient perceived quality of care (35, 44, 48). Additionally, relational coordination between providers and family members was associated with family members' preparation for caregiving (44) as well as shared decision making with the patient (49).

Shared information systems are another structure that has been shown to reduce barriers to communication, thus strengthening relational coordination and subsequent performance outcomes (28, 29). Shared information systems are expected to support coordination when they are accessible to all stakeholders, provide visibility to the work process and used as a supplement rather than a replacement for other forms of communication (50), the research findings have shown mixed results across industries. In healthcare settings, shared information systems have been positively associated with relational coordination among care providers (36, 41). Other healthcare studies suggest that relational coordination may decrease challenges associated with lack of proximity in patient portal networks (51). Testing this theory on the introduction of clinical information systems in chronic care delivery, Cramm and Neiboer (36) found a strong correlation between the existence of clinical information systems and measures of RC as reported between clinicians. We are extending this theory to the relationship between telehealth and teams formed between clinicians and informal caregivers. In this paper we connect RC, an evidence-based framework and management tool, to the production of healthcare in informal teams composed of patients, providers, and caregivers.

## Materials and Methods

This single-case study aims to understand how increased use of telehealth during the COVID-19 pandemic impacts caregiver perceptions of the dimensions of relational coordination. This paper uses autoethnographic methods to study the lead author's experience as a caregiver of her daughter who experiences hEDS. Research for this paper began before the COVID-19 pandemic, at which time the aim was to study relational coordination between the caregiver/lead author and several providers involved in managing the caregiver's daughter's hEDS. This initial work was subsumed into this current research project, which was re-directed to study how relational coordination between the caregiver and providers was impacted by increased use of telehealth during the pandemic.

Yin (52) argues that holistic single-case studies are appropriate in critically testing a well-formulated theory that has a set of propositions and conditions under which the prepositions are supported or hindered, as is the case with the theory of relational coordination. This method is also particularly helpful in understanding a rare, “extreme,” or otherwise exemplary case (52). In this instance the caregiver is a Ph.D. researcher, and a highly involved mother of a daughter with a rare genetic disease that requires chronic pain management. Thus, this single case study is intended to be a deep dive into the experiences of an exemplar for the purpose of extending literature on relational coordination theory to the process of caregiving using telehealth.

We used autoethnographic methods to determine the relationship between telehealth use and care coordination using relational coordination theory between the informal caregiver and the provider team. As a qualitative approach to research, autoethnography aims to describe and systematically analyze one's personal experience in an effort to understand a broader culture (53). In line with Jones' (54) definition of autoethnography, our first commitment is to explore the “dynamic relationship between theory and story” [p. 231]. Here, the corresponding author uses personal accounts reflexively to shed light on the broader context in which her experiences have occurred (55). While criticisms of this approach have centered on its “rampant subjectivism” [(56), p. 48] and lack of rigor, others have noted the benefits of integrating story and social science, bridging creative, and critical aspects of inquiry (57). Others argue that autoethnographies can be rigorous when systematically designed with well-defined research questions that allow them to be inclusive of personal and social phenomena (58), as is the case with this paper. We believe that subjectivity is a strength of this paper, allowing for more detailed and holistic observations that would not be possible if the researcher was removed from the subject.

In this study, autoethnography was accomplished through reflective journaling and participant observation by the lead author regarding her experience as a caregiver and the relational coordination that she experienced with members of her daughter's care team. Reflective journaling was used to determine (1) the tasks performed by the caregiver, and (2) how the caregiver's experience of relational coordination with providers changed as her use of telehealth increased during COVID-19 pandemic. The caregiver further recorded a count of the remote and in-person interactions that she had with members of the care team before and during the COVID-19 pandemic. In several instances the author also documented segments of conversations she had with her daughter, the patient, as well as conversations with other members of her daughter's medical and social care teams during the COVID-19 pandemic. This journaling was supplemented with information from provider notes to confirm and elaborate clinical observations.

This research also involved several interactive interviews to provide in-depth account and understanding of the participant's lived experience (59). Development of the interview questionnaire was iterative and began with the second author developing a set of interview questions to illuminate the experience of the caregiver before and during the COVID-19 pandemic. These questions were largely divided into (1) questions about the process of caregiving using telehealth and in person visits, and (2) experience questions that asked the caregiver to explain how the shift from in-person to predominantly remote care impacted the seven dimensions of relational coordination (frequency, accuracy, and timeliness of communication, as well as the ability to problem solve, share goals, share knowledge, and develop mutual respect).

Interviews were completed after the start of the COVID-19 pandemic, allowing for a post-intervention assessment. These interviews occurred as a collaborative endeavor between the participant researcher (corresponding author) and a second researcher (co-author). The interactive interviews occurred multiple times throughout the COVID-19 pandemic and were situated in the context of a well-established working relationship between the two researchers. Content analysis was performed by both the primary and secondary researchers to capture both the emic and etic perspectives on the transition from in-person to remote caregiving (60).

## Results

Table 1 provides summary data comparing telehealth visits between 2019 and 2020. During the period between March-December, 2020, the informal caregiver used telehealth to attend several visits with a specialist and primary care clinicians (see Table 1). Also shown are comparison data from the same months in 2019. This data shows a substantial increase in telehealth visits from 2 telebehavioral visits in 2019 to 54 visits across specialties in 2020. We see that the total frequency of visits (telehealth + in-person visits) was greater during 2020 than in 2019. This trend is also reflected at the individual clinician level.

**Table 1:** Comparison of number and type of in-person and telehealth visits, March–December 2019 and 2020, for a pediatric patient with hypermobile Ehlers-Danios Syndrome (hEDS).

	**March 2019**–**December 2019**		**March 2020**–**December 2020**	
**Provider type**	**In-person visits**	**Telehealth visits (phone, video)**	**In-person visits**	**Telehealth visits (phone, video)**
Primary care physician	3	0	1	5
Therapist	8	2	9	10
Genetics	1	0	1	1
Functional medicine	0	0	0	3
School nurse	5	0	6	3
School counselor	52	0	16	22
Physical therapy	0	0	1	1
Psychiatry	0	0	0	1
Case manager	0	0	0	3
School IEP team	2	0	0	5
Totals	**71**	**2**	**34**	**54**

*Bold values represent total number of visits for each type of visit for each time period*.

The caregiver used Zoom, Doximity, and her cellular phone (iPhone 10 with messaging, audio, and FaceTime capability) to communicate with providers remotely. The fact that visits could be done remotely increased flexibility that facilitated timely interactions and saved the patient and the caregiver time commuting, and made it possible for the caregiver to avoid having to schedule and pay for childcare and other expenses associated with travel, and as a result, the frequency of appointments was higher during the COVID-19 pandemic as compared to pre-pandemic levels.

### Informal Caregiver Role

Our findings reveal the unique role of an informal caregiver of a pediatric patient with a chronic, multisystem condition, and their perspectives on care coordination in the context of 54 telehealth visits between March and October 2020. During the study period, the informal caregiver allocated ~20–24 h per week coordinating care and services for her daughter.

Caregiving for a patient in this case was found to involve four interrelating roles; logistics support, boundary spanner, home health aide, and cultural translator. Perhaps the most visible role was that of logistics support. The caregiver was responsible for scheduling visits, transporting the patient, and collecting medical supplies to support the patient's treatment plan (see Table 2).

**Table 2:** Coordination role of informal caregiver for a pediatric patient by coordination area: pain management, academic accommodations, mental health support, and future planning.

**Coordination area**		**Relevant providers**	**Coordination activities**	**Exemplar**
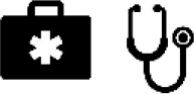	Pain management	Primary care physician (PCP), orthopedic gastroenterology, etc.	Schedule and prepare^a^ for appointments; implement adjustments to care plan and home care, including medication management. Respond to acute medical needs, including emergency services.	Share new peer reviewed studies with PCP related to hEDS treatments and pain management methods which serves as the basis for discussion at the next appointment.
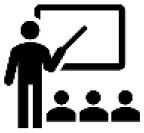	Academic accommodations	School teachers, counselor, physical therapist, school nurse, PCP	Prepare for, attend and follow up on Individualized Education Plan (IEP) meetings and progress reports; provide school team with updates from medical team; problem-solve challenges that come up and which impact academic work, e.g., fatigue, pain, anxiety, etc.	Proactively reach out to core team members to share updates to plan of care, including recommendations from specialists. Schedule phone calls with individual teachers who are unable to attend team meetings to share knowledge. Send emails with home updates related to pain management.
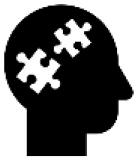	Mental health support	Therapist, PCP, music teacher, school counselor	Schedule and prepare for therapy sessions, including parent huddles to provide updates and debrief sessions and to iterate the plan of care.	Access private music instruction and opportunities outside of school district. Initiate discussions with multiple teachers and leaders in the school district about how to provide more supports around music and arts as a form of expressive, socioemotional learning.
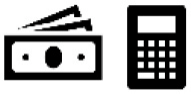	Future planning	Genetics, PCP, school, financial advisor, insurance company	Schedule, attend and follow up on meetings with financial advisor; engage with case manager to optimize access to health benefits; engage in genetic counseling and testing to identify risk factors that have potential to impact future financial and healthcare plans.	Identify, access, and engage financial planner to provide guidance on long term financial needs based on current health information and future health needs, including genetic testing results; conduct in-depth research on healthcare plans to meet anticipated future needs.

a *Appointment preparation includes, but is not limited to, uploading/emailing/photocopying visit summaries or notes from other provider appointments/meetings, developing a list of discussion items, and/or questions that have come up since last appointment and having a conversation with the patient regarding the appointment itself, what to expect and any concerns she may have*.

The second role was that of boundary spanner between providers and teams. The caregiver spent large quantities of time doing things like printing/scanning/emailing information and sharing it within the team. She shared perspectives between team members, interpreting clinical findings and helping to facilitate shared knowledge. One example where this action was particularly important was after her daughter received her yearly genetics exam. The caregiver shared the up-to-date information with other providers and specialists which greatly impacted their recommendations for modifications to her treatment plan moving forward.

Third, the informal caregiver performed several functions that would otherwise be the responsibility of a home health aide. This includes helping with activities of daily living before and after school, preparing special diet and nutritional supplements, assisting with pain management strategies, and responding to acute situations that come up unexpectedly, such as GI distress, joint pain, body temperature regulation, and anxiety related to these symptoms.

The fourth role was social translator. In this role, the informal caregiver was responsible for bridging the clinical sphere with the patient's home sphere and is captured in the following quote:

*During health visits, I help to bring up and talk through aspects of my daughter's life that are either barriers to treatment, or potential opportunities to improve management. I help my daughter to make decisions about adherence to a particular treatment strategy or practitioner. Even now, several years into the disease, we regularly discuss the many possible interventions and lifestyle modifications to iterate a treatment plan that will add the most value to my daughter's life*.

-Informal Caregiver

### Impact of Telehealth on the RC Dimensions

The change from in-person to remote visits initially impacted measures associated with communication more than measures associated with relationships. More broadly, the option for remote visits allowed team members to spend more time talking, problem solving, and coordinating.

#### Timeliness

The most cited communication change observed during the study period was the change to the timeliness of visits. As previously discussed, the challenges around travel and child care were virtually eliminated with the transition to remote visits, allowing the caregiver to move quickly to book the first appointment with the physician that was available. This dramatically increased the timeliness of communication, allowing the patient and caregiver to iterate treatment plans at a much greater speed and is reflected in the following quote:

*Before COVID-19, we would have to schedule an in-person visit with the PCP to discuss changes to the plan of care. Now we can hop on the phone or video call to talk through changes in status and/or responses to specific interventions, including next steps for care. Something that used to take months now takes no time and we don't have to figure out childcare for our younger children or account for travel time*.

–Informal Caregiver

This was particularly impactful for multi-appointment initiatives where a meeting with the PCP indicated a need to see a specialist, which often required a follow up visit with the PCP to work the specialist's suggestions into the patient's treatment plan. Instead of multiple in-person visits, telehealth provided a mechanism for a quick follow-up and debrief of specialist appointments. Likewise, timeliness of communication between the informal caregiver and the therapist improved with access to telehealth services and is captured in the reflection below:

*Telehealth appointments have also occurred (weekly) between her therapist and informal caregivers (my husband and I). This has been incredibly valuable in saving time before and after in-person therapy appointments to communicate updates and debrief how sessions play out. The coordination with the therapist has improved greatly through the use of telehealth services – it's easy to schedule these appointments and they've morphed into more of a “huddle” now that we've been doing it for a few months- a time for us to sync up, provide highlights and for the therapist to suggest the next course of action. Before, we used to go into therapy sessions and I would do my best to provide an update in a short amount of time, unsure of what information would be most helpful to communicate to the therapist and trying not to talk about it in front of my daughter – and also trying to save time so that she could get the most of the time. Now, the therapist is able to count our “parent telehealth meetings” as appointments, bill for them and we aren't rushed. Also important is that we are not in front of my daughter while we're talking. This has provided us with the space and time to build shared goals and co-produce a course of treatment for my daughter. It's extremely efficient and feels aligned and collaborative*.

-Informal Caregiver

Overall, the remote care appointments were used for follow up to in-person visits and to seek advice when adapting a treatment plan to the constraints of the patient's life. Using telehealth to provide opportunities for coordination to support the in-person clinical procedures reduced the number of duplicative visits and improved timeliness of care.

#### Frequency

The frequency of visits with the care team increased during the pandemic as a direct result of the option to schedule remote visits. This reduced the time allotted for the informal caregiver to attend remote visits from several hours to ~30 min. The increased frequency of care delivery using telehealth during the COVID-19 pandemic allowed the caregiver to move more quickly and build momentum with different interventions.

*We've had more communications with our daughter's PCP and therapist specifically since COVID-19. Part of this is due to the ease at which we are able to schedule telehealth appointments versus in person appointments. Also, providers can now bill for phone and web-based appointments so they have blocks of time carved out to touch base with patients who need the extra support. More frequent communication, especially with the PCP and therapist, has resulted in more efficient communications that take less time overall*.

-Informal Caregiver

The decrease in time commitment (finding child care, driving, etc.) required for each individual visit made it possible to meet with specialists and the PCP in the same week to incorporate the new insights into the treatment plan. For instance, during one appointment, our PCP recommended a medication consultation with a specialist. That same day, the PCP coordinated the consultation and within the same week, we had met with the specialist via telehealth to discuss medication management. This type of iterative meeting schedule is common in the treatment of patients with hEDS. Telehealth made it possible for treatment to be iterated in a much more condensed manner, allowing the team to meet a specific goal in a matter of weeks, where it would previously have taken months.

#### Accuracy

The adoption of telehealth both positively and negatively impacted the accuracy of communication. The limited window and two-dimensional view offered in video applications made it challenging for the participants (patient, caregiver, and provider) to observe body language, which limited the perception of social cues. Physical exams, orthopedic tests, and other sight based clinical procedures often had to be repeated in-person or were not attempted remotely. This is consistent with the notion that telehealth is not appropriate for some procedures.

Interestingly, the frequency and timeliness of communication appeared to positively impact the accuracy of communication being shared, though this appeared to be a secondary outcome. This was especially true in the case where the caregiver was responsible for transferring information between providers. The fact that meetings with the primary care provider could happen in the same week as a meeting with a specialist meant that the information from the specialist was fresh in the caregiver's memory, which facilitated accuracy of information transfer. Additionally, having the option for brief follow up calls with providers was an opportunity to clarify details and next steps:

*The ability to follow up with healthcare providers after an appointment has been very helpful. We recently had an in-person genetics appointment which was followed by a telehealth appointment to clarify next steps for genetic mapping, physical therapy, and at-home management of symptoms. For anyone who has attended a genetics appointment, even the most educated among us can be easily overwhelmed by the technical details communicated by these providers, making follow up appointments critical to clarifying important details about the plan of care*.

*During another telehealth appointment, the PCP coordinated with a specialist to gain up-to-date information on a pain medication. These follow up telehealth calls have allowed us to update and implement the plan of care more efficiently and effectively*.

-Informal Caregiver

In some instances, the caregiver was able to organize phone calls or video-conferences where both specialists and the primary care provider attended the meeting, which also increased the fidelity of information sharing between groups. As a last point, the option to meet with providers remotely made it possible to meet with new specialists who were too far away to visit in-person. This made it possible to access providers with more specific knowledge about the condition.

#### Problem-Solving

Telehealth provided the space and opportunity for more problem solving communication. As previously discussed, telehealth works for some clinical needs and not for others. The increased frequency of appointments gave the caregiver more time with providers, allowing for a shift from information sharing to problem solving communication. By comparison, the caregiver described pre-COVID in-person visits as being quick, unidirectional, and clinically oriented to facilitate sharing large amounts of information in discrete windows of time.

The convenience of telehealth allowed the patient and the caregiver to have a follow up appointment with the geneticist after the annual in-person exam. The additional time made it possible to solve problems related next steps for genetic mapping, school services, physical therapy, and at-home management of clinical symptoms. Similarly, the caregiver began scheduling remote visits with her daughter's therapist to debrief and communicate updates between sessions:

*Before COVID-19, we'd have to hope that there was a few minutes in between patients for us [caregivers] to provide any updates to the therapist. The same issue happened at the end of the appointment. Sometimes we wouldn't have time to debrief with the therapist and would have to figure out how to have a quick conversation before the next appointment. It didn't always happen. Since COVID-19, we've scheduled parent huddles between our daughters' appointments. They aren't rushed but also aren't very time consuming and allow us to problem solve around specific challenges that come up. We've seen more progress since the parent huddles*.

-Informal Caregiver

#### Shared Knowledge

The increase in frequency of visits created more opportunities to share knowledge between the caregiver and providers, which impacted both the volume and quality of information shared. This increase in communication impacted the provider/caregiver dyad, but also impacted the ability for providers and other members of the team to share information through the caregiver as a boundary spanner:

*There's just been more time to connect with providers related to day-to-day management of hEDS symptoms and our daughter's response to specific interventions. It also seems like our providers have more time to understand the daily impacts of the disease. I'm not sure if that's because they aren't spending time on other things and have more time to spend with patients but there's definitely been a shift. Our PCP, therapist and other specialists appear to be less rushed and have more capacity to coordinate with us and other providers*.

-Informal Care Provider

#### Shared Goals

Goal setting for the patient is iterative and is driven by annual genetics, PCP and therapy appointments, which provide data and insight for how to prioritize care. Additional facetime between the caregiver and providers gave them space to discuss these goals and how they impact and are impacted by the treatment plan. What's more, this additional time allowed the caregiver to work with the primary care provider to come up with strategies to align these goals with the goals of the patient. From a more clinical perspective the extra time allowed the caregiver to work with the primary care provider to synthesize and prioritize the various goals of the clinical specialists. This is especially important in the case of multi-systemic diseases like hEDS, where attending to all of the goals of each specialist (cardiologist, orthopedist, geneticist) is not realistic or feasible.

#### Mutual Respect

Mutual respect between the informal caregiver and providers remained largely unchanged during the transition to telehealth visits, as indicated by the following quote:

*There's a high level of mutual respect that hasn't changed since COVID-19, but a higher quality of communication has emerged and it's strengthened our shared goals for the patient, our daughter*.

-Informal Caregiver

hEDS is associated with a wide range of other co-occurring diagnoses, which are often eclipsed by the principal diagnosis. Reporting symptoms of the patient, which interventions are working, and which are not, is important to our understanding of the disease pathology. The frequency of interacting with providers, especially the primary care provider, allowed a lot of the more subtle observations of the caregiver to be fleshed out in full. Additional time only amplified the pre-existing willingness of the PCP to help the caregiver think through how best to manage symptoms and co-produce a treatment plan:

*The uncertainty of the path of treatment felt okay because we were navigating these uncharted waters together*.

-Informal Caregiver

## Discussion

U.S. national and state level policies during the COVID-19 transformed the landscape of healthcare payment and delivery in two important ways. First, providers were authorized to conduct telehealth visits which were reimbursed at parity with in-person visits (1, 20, 63). Additional payment structure changes allowed providers to utilize check-in codes for telehealth visits delivered via phone, web-based platforms and/or email communication (64). Second, the Office of Civil Rights provided flexibility around HIPAA laws with respect to patient privacy and confidentiality in accessing telehealth services (18). This offered opportunities for providers to communicate with patients and/or caregivers via phone and/or other less-secure mechanisms. The healthcare payment and service delivery policy changes have impacted the ways in which patients and caregivers interact with providers and in some cases, has reduced barriers to accessing services which has resulted in a more efficient and effective plan of care.

This study investigated the care coordination of a pediatric patient in Massachusetts with a complex chronic condition from the perspective of her mother, an informal caregiver, in the context of the healthcare policy changes to telehealth service delivery. Overall, communication and coordination were observed to have improved over the course of the study period, allowing for the patient plan of care to be implemented and adjusted more efficiently and effectively. These findings extend the current body of research by pointing to the importance of care coordination and the critical role of relational coordination in the provision of care. Specifically, our findings support the notion that productive collaboration between informal caregivers and healthcare providers is likely due to a combination of communication frequency, accuracy, problem solving, and timeliness supported by shared goals, shared knowledge, and mutual respect (48).

We interpret these findings around care coordination between the informal caregiver and healthcare providers in several ways. First, we view them from a broader context by recognizing the informal caregiver as a co-producer of patient health and well-being. Because more medical care is provided at home than in formal health care settings, there is a need for informal caregivers to be viewed as formal members of the health care team (44). Arguably, this may be more important for patients who have rare, complex diseases for which shared knowledge from the caregiver is crucial to assessing the patient and developing and adjusting the plan of care. Thus, the increase in timeliness and frequency of communication in addition to shared knowledge via telehealth visits between the caregiver and providers is important in the shared planning and execution of care (65).

Second, accessing telehealth services through video conferencing and phone calls highlights the importance of shared information systems in the delivery of health care services. In the case of this study, the shared information systems included a web-based platform for engaging in video conferencing and use of phone lines. Together these supplemental forms of communication reduced barriers to access to care and supported key components of relational coordination. This finding supports the structure/process/outcomes model of relational coordination proposed by Gittell (45).

Third, this case study provides insight into several caregiving functions that have been impactful in the care for this patient. A resounding theme of the caregiving role is that of boundary spanner and coordinating work among providers (66). These findings concur with a growing body of literature that highlights the need to develop and study tools to support caregivers as boundary spanners (47, 67). This role can be further augmented with management tools, like stakeholder charts and information sharing platforms offered by telehealth that are designed to help caregivers accurately and effectively coordinate care (68). That being said, building checklists for patients, like other means of explicit accountability, is a double-edged action. While it can give welcomed structure to caregivers who are able and willing to provide care, it has the potential to lock other caregivers into roles that they can't perform or are inappropriate. In the case of caregivers who are already receiving social pressure to give labor to the patient, this can put additional strain on that relationship, which has the potential to create unintended consequences for the health of the caregiver (69) and the quality of care provided to the patient (70). Gage and Albaroudi (71) instead argue that an appropriate tactic is to measure the capability of caregivers and co-produce one or more responsibilities according to the characteristics of their specific involvement. From a practical standpoint, it may be helpful to equip caregivers with the knowledge, skills, and abilities needed to prepare for appointments, utilize time during appointments and follow up in between appointments.

We highlight policy and practice implications including continued access to telehealth services and systematically assessing the strength of relational ties between caregivers and providers. Caregiver access to telehealth through videoconferencing, phone and/or email communications has potential to improve care coordination and result in more efficient and effective implementation of the plan of care. Providers may consider increasing the adoption of newly created check-in codes (64). This incentivizes the use of shorter visits that can be synchronous (phone or video) or asynchronous (secure message). This nimble medium for coordination of care increases timeliness and frequency of communication without requiring the time or financial expense of a full-length visit. This study found that check-in and other billing codes were often used to reimburse providers for interactions with caregivers. This practice requires support of insurance payers and health systems in the form of explicit policies indicating that providers can bill for the time that they spend with caregivers. This limits uncertainty around what actions are billable and allow providers to feel confident that their time spent communicating and coordinating with caregivers is within their scope of practice and will be reimbursed.

Additional research is needed to understand the generalizability of the findings of this single case study of an exemplar. Subsequent studies with larger sample sizes are needed to assess the relationship between telehealth and care coordination between providers and caregivers for the treatment of complex patients. Relational coordination, in particular, can be measured quantitatively through the use of the validated Relational Coordination Survey, which makes it possible to do a follow up survey to determine generalizability (29, 72). It may also be valuable for providers to ask open-ended questions to informal caregivers related to goal setting and treatment plan strategy, something that can further increase patient value (73). This paper links telehealth to process measures (relational coordination, co-production, goal setting, etc.). Future research is needed to connect these process measures to performance outcomes, such as hospitalizations, health measures, and other measures of cost of care. Making this connection from process to outcome enhance our understanding of how the discussed practices impact the efficiency and effectiveness of care.

This paper focused on how telehealth impacted the caregiver's ability to provide care in their role as a boundary spanner. Research is needed to understand how this support impacts caregiver burden, which has been associated with caregiver anxiety and depression in other contexts (74, 75). A secondary benefit of this investigation is that we found that telehealth reduced time and expense of caregiving by decreasing travel and the need to coordinate childcare. Additional research is needed to explore these findings more rigorously and understand how telehealth impacts the cost of caregivers for rare disease. These findings will help insurance organizations to understand the value of caregiving, which will inform a conversation about caregiver compensation and other forms of support for the caregivers.

From a broader systems perspective, this case study suggests that the use of telehealth services are a mechanism to facilitate and support high quality relationships between providers, caregivers, and patients. Particularly for patients with chronic conditions, the convenience of telehealth services for clarifying important details in the plan of care, medication management and problem solving around specific interventions offers the potential for timelier implementation and/or iteration of the care plan. Implementing telehealth in this way has the potential to support the caregiver's role and simultaneously reduce caregiver burden. We do not suggest telehealth as a solve-all solution for improving care coordination. It must be aligned with broader efforts to build team performance and add value for patients and caregivers. To that end, healthcare providers seeking to utilize telehealth services as a supplement to in-person visits and to improve care coordination may benefit from relational coordination training and other methods to build collaborative team cultures. Within this context, training may focus on communication and facilitation skills as well as building shared goals, shared knowledge and mutual respect within and across stakeholder groups. Training may also include best practices for coordinating with caregivers, creating care planning guidelines, understanding and respecting caregiver's preferences and capacities and developing shared goals for the patient.

## Conclusion

The unique role of the contributing author as both participant in her daughter's medical care and member of the social community has put her in the position of being able to bridge these two cultures, identify opportunities for them to work together, and point out misalignments.

This study has several limitations. While the autoethnographic style helped to nest the observations directly in the lived experiences of the corresponding author as an informant without interpretation, it introduced a specific type of researcher bias where there is no second party to check her portrayal of a desired outcome. That said, it can be assumed that her inherent bias is at least in part influenced by her lived experience, making it a signal in its own right. Further, the collaboration with a second researcher helped this study to be carried out systematically, using multiple methods to inform the analysis.

Perhaps more impactful are the limitations to sample size of one patient and external validity. The corresponding author is a highly educated researcher and confident in her ability to correctly interpret and explain information. She understands the pitfalls inherent in patient/provider power dynamics, and knows how to advocate for herself and for her daughter. Moreover, she has the capacity to devote time and the resources to her daughter's care. These limitations speak to a larger challenge of variation between informal caregivers, which affects their abilities to perform a single defined set of functions. Moving forward, this means that support and measurements of quality must be broken down into specific caregiving functions, allowing caregivers to define the bounds of their contribution. This itemization can maximize performance of individual tasks, while minimizing pitfalls associated with assuming caregiver ability.

## Author's Note

The goal of this research is to understand where telehealth adds value for patients and their families to inform policy that expands access to remote health care. Because the corresponding author is the instrument in this qualitative study, it is beneficial to provide a brief explanation of the researcher's positionality as it relates to the research (61, 62). She holds a Bachelor of Arts degree in psychology, a Master of Public Administration and a Ph.D. in Social Policy–an interdisciplinary background that has informed a research agenda centered on organizational change and relational practices that support high performing teams, organizations and communities. Using both quantitative and qualitative approaches, she has studied team dynamics in multiple healthcare contexts. Her familiarity with theories and frameworks that support high performance, such as relational coordination, have provided the foundation for which the analysis in this study is based on. The author's perspective as an informal caregiver is also informed by her positionality as a privileged white female with high digital literacy.

The second author is a Ph.D. candidate in Social Policy, and is simultaneously working on a Master's in Business Administration. He studies caregiving and patient adoption of telehealth. The second author's perspective on both topics is informed by his positionality as a white male with substantial privilege and high digital literacy. His background and training in social policy has helped to inform a perspective that telehealth has the potential to be beneficial as a supplement communication medium to in-person visits for specific procedures. These benefits, however, are lost on those who lack the technology, internet bandwidth, and/or high digital literacy needed to effectively make use of the technology. This framing has led him to explore different contexts for telehealth use to identify strengths and weaknesses of the medium.

## Data Availability Statement

The raw data supporting the conclusions of this article will be made available by the authors, without undue reservation.

## Ethics Statement

The studies involving human participants were reviewed and approved by Brandeis University Institutional Review Board.

## Author Contributions

The unique role of the corresponding author as both a social science researcher and participant in her daughter's medical care and member of the social community has put her in the position of being able to bridge these two cultures, identify opportunities for them to work together, and point out misalignments to inform research, policy and practice. BK contributes his knowledge and expertise in qualitative methods, healthcare coordination and delivery and informal caregiving to systematically understand and evaluate value-driven approaches to care. All authors contributed to the article and approved the submitted version.

## Conflict of Interest

The authors declare that the research was conducted in the absence of any commercial or financial relationships that could be construed as a potential conflict of interest.

## Publisher's Note

All claims expressed in this article are solely those of the authors and do not necessarily represent those of their affiliated organizations, or those of the publisher, the editors and the reviewers. Any product that may be evaluated in this article, or claim that may be made by its manufacturer, is not guaranteed or endorsed by the publisher.
